# Exploring Teachers' Back Pain Concerning Their Habits, Rules, Leisure Activities, and Physical Activity Breaks at Work

**DOI:** 10.1177/00469580211060256

**Published:** 2021-11-23

**Authors:** Katarina Galof, Lea Šuc

**Affiliations:** 1 Department of Occupational Therapy, Faculty of Health Sciences, 37663 University of Ljubljana, Ljubljana, Slovenia; 2 The University Rehabilitation Institute Republic of Slovenia, Ljubljana, Slovenia

**Keywords:** workplace design, ergonomics, academic staff, health, professional habits, and rules

## Abstract

The role of a teacher in the Faculty of Health Sciences involves teaching students, preparing lessons, and participating in other activities such as research projects and management processes. Professional participation is part of their involvement in daily occupations, which are taking place in teachers’ socio-cultural context and are necessary for their well-being. Teachers' work performance can be enabled or constrained by their professional habits, including habits while using computers. We investigated awareness of and adherence to recommendations on ergonomics and preventive measures for back pain among the Faculty of Health Sciences employees. This study was aimed at investigating the relationship between the influence of their habits during computer use and back pain. An online questionnaire was sent to the teaching staff of the Faculty of Health Sciences (*n* = 115). 73% of the staff opened the online questionnaire, 43% fully completed the questionnaire. Data were processed using SPSS statistical program, version 20.0. Descriptive statistics, Pearson’s correlation coefficient, and factor analysis were calculated. Analysis of the results showed a significant statistical association between the professional habits and roles [daily computer use (*r* = .443, *P* < .01); position of different body parts during computer use [head (*r* = .669, *P* < .001), shoulder (*r* = .446, *P* < .01), legs and feet (*r* = .483, P < .01), screen inclination (*r* = .577, *P* < .01), adjusting chair settings (*r* = .608, *P* < .01), distance between eyes and screen (r = .766, *P* < .01)]; physical activities break at work [*r* = .758, *P* < .01], and back pain. Considering ergonomic principles when designing the work environment can have a major impact on employees' health and quality of work. As experts in the field of health sciences, faculty members are not sufficiently aware of the impact of an improperly designed work environment on employee health, which affects work habits.

**What do we already know about this topic?**

Previous studies have established the association with musculoskeletal disorders and pain in various human activities.

**How does your research contribute to the field?**

This study examines teachers' perceptions that burnout is responsible for their health.
It also examines the relationship between achieving health, well-being, and participation in life through engagement in leisure activities.

**What are your research’s implications toward theory, practice, or policy?**

The findings can help management understand the needs and aspirations of teachers, which was particularly important in Slovenia last year. (During the Corona period, all faculty teachers worked from home year-round). Management encourages them to create a better work environment and, based on the results, can create a recovery plan for workers with long-term computer use to prevent burnout.

## Introduction

According to the WHO, approximately 1.71 billion people worldwide suffer from musculoskeletal disorders. Among musculoskeletal disorders, low back pain causes the greatest burden with a prevalence of 568 million people. Musculoskeletal disorders are the leading contributor to disability worldwide, with low back pain being the leading cause of disability in 160 countries (WHO). Musculoskeletal disorders severely limit mobility and dexterity and lead to early retirement, decreased well-being, and reduced ability to participate in social life. Due to population growth and aging, the number of people with musculoskeletal disorders is increasing rapidly. Disabilities related to musculoskeletal disorders have increased and are projected to continue to increase in the coming decades.^1^

The International Association for the study of pain^2^ defines pain as an unpleasant sensory and emotional experience associated with or resembling actual or potential tissue damage. They also highlight 6 important clues and etymology: (1) Pain is always a personal experience, influenced to varying degrees by biological, psychological, and social factors. (2) Pain and nociception are distinct phenomena. Pain cannot be derived from sensory neuron activity alone. (3) Humans learn the concept of pain through their life experiences. (4) It should be respected when a person refers to an experience as pain. (5) Although pain usually serves an adaptive function, it can have a negative impact on function and social and psychological well-being. (6) Verbal description is only one of several behaviors used to express pain. The inability to communicate does not preclude a human or nonhuman animal from feeling pain.^2^

Humans adapt to sitting with varying limb positions, while the body position remains relatively stable. Problems of the musculoskeletal system first manifest themselves as functional disorders. If they are not detected and corrected in time, morphological and structural disorders may result.^3-5^ A body position in which we remain for a long period can cause static stress on muscles and joints. Some factors cause the susceptibility of the spine to the occurrence of discomfort, pain, and injury.^5,8,9^

The occurrence of pain and factors associated with musculoskeletal disorders have been studied in employees working in various fields of human activity, including Slovenian physical education teachers, with the most frequent health problems occurring in the lower back in both genders.^8^ The results of a study in the Netherlands^9^ showed that workers who perform heavy physical work and workers who are mainly sedentary have problems in the neck and upper extremities. Pain and musculoskeletal disorders occurred due to irregular, non-ergonomic sitting, flexion and rotation of the neck during work. A statistically significant difference between the occurrence of lower spine pain in teachers and the time the body is in the position of static flexion was demonstrated by Wong and his colleagues.^10^ Sleep disturbance, prolonged standing during the session, and irregular physical activity have been associated with neck/shoulder pain and headache.^6^ Durmus and Ilhanli^11^ found that physical activity is associated with low back pain, especially among teachers in Physical Education classes.

Many individual factors (genetics, age, gender, anthropometrics, psychosocial profile of an individual, cognition, and physiology), physical environment and biomechanical factors (strength, posture, movement, and vibration), and work organization are potential risk factors in adult computer users that influence musculoskeletal disorders.^6,12,13^ Slovenian researchers^14–20^ investigated the problem setting Slovenia between Slovenian workers in different work settings. Bilban and Djomba^14^ focused not only on the type of work condition, but also on age, gender, lifestyle, education, social class, living environment, and psychological well-being as risk factors for the development of back pain. However, the importance of the influence of lifestyle with physical activity on the occurrence of back pain should not be forgotten.^21,22^ All employees should learn how to work properly and protect their health, including professional habits and roles in routine workplace activities. Employees should take responsibility for their health at work, as we spend one-third of our daily time at work.^13,17^ Ergonomic promotion and interventions to improve and maintain health in the work environment are successful only when employees actively collaborate with professionals to reduce stress and improve work-related health problems.^23^ Consideration of work-related factors and individual factors such as female gender, age, and teaching experience^6^ is also important in reducing stress. Poor posture, unsuitable furniture, lifting, and carrying have also been associated with a high prevalence of musculoskeletal disorders.^4^ Regular physical activity has been shown to be a protective factor.^4^ For example, a recent study of school teachers in Botswana found that musculoskeletal disorders prevented some teachers from performing their normal activities and caused some to change jobs or duties, limit their activities at home, and seek medical attention.^4^

The Slovenian National Institute of Health^24^ collected data on absenteeism and reported that 4.5% of absenteeism in Slovenia in 2020 was due to back pain, which is one of the most common causes. As health professionals, we should believe that we alone are responsible for our health. As health professionals, we have the knowledge to change workplaces and reorganize our professional habits and roles. The aim of our study is to find out whether workers receive recommendations on the principles of ergonomics in sedentary work, whether they engage in physical activity in their free time, how often pain symptoms occur, and whether there is a relationship between these items. The results of the study will guide the management in designing workstations and the employees in finding human resources in the Faculty of Health Sciences. The findings will also help in formulating appropriate intervention targets and management strategies to improve teachers' health in performing their professional duties and habits, minimize the prevalence and impact of back pain among teachers, and reduce absenteeism.

## Methods

### Participants and Data Collection

The anonymous online survey was emailed to first-cycle and second-cycle teachers (pedagogical staff) (*N* = 115), employees of the Faculty of Health Sciences, University of Ljubljana, in early 2020, before the government declared the COVID-19 epidemic. We sampled 48.3% of the teaching staff from the fields of midwifery, occupational therapy, physiotherapy, dental prosthetics, orthotics and prosthetics, radiology technology, sanitary technology, and nursing who completed the questionnaire. 14% were men and 86% were women.

### Measurement Instrument

The increasing amount of daily paperwork increases the amount of sedentary computer work among teachers. Based on a review of the literature and related laws in this area, we formulated individual statements of the questionnaire. We pilot tested the questionnaire with 20 elementary school teachers to clarify the individual statements of the questionnaire. The online survey was designed to collect information about the design or setup of the workstation for completing computer tasks (posture and positioning of the teacher’s work), sitting time, frequency of pain symptoms, and physical activity during leisure time. Reliability was measured using the Cronbach’s alpha coefficient for 8 items on posture of different body parts while working at the computer (*α* = .97). Two of the items had lower correlations with the overall reliability coefficient score, but their exclusion did not improve the reliability coefficient and therefore were not excluded. Validity was assessed using the correlation of the same variables. The value obtained was 48 DF (.05) = .273, with Pearson correlation coefficients ranging from .486 to .694 (*P* < .05).

The first part of the online survey included demographic data (gender, age, AND education level) and occupational data (years of service, daily hours worked on computer tasks, preventive rest breaks at work/active rest breaks at work/physical activity breaks), as well as teachers' subjective perceptions of the frequency of pain symptoms. If pain interfered with teachers' daily computer work, they rated the frequency of occurrence of back pain on a five-point Likert scale, with 1 representing once per week and 5 representing 5 times per week. In the second part, teachers rated the postures and positions they adopt while working on the computer and the work environment in which they use the computer. The workplace is designed or set up in such a way that computer tasks can be performed. In formulating the questions, we considered work postures in light of the ergonomic recommendations for sedentary computer work.^25^ We asked teachers to rate the factual statements related to the ergonomic recommendations at their own workstation by rating the posture, chair, keyboard, and monitor on a 5-point Likert scale from 1 to 5, with 1 representing “strongly disagree” and 5 representing “strongly agree."

In the third part of the online survey, we asked teachers how they spend their leisure time and what kind of lifestyle they have. We were interested in how often they do physical activities in nature in their free time, or how often they do physical activities in different sports centers or gyms. We offered them the following response options: regularly every day; every other day; at the end of the week; occasionally when I feel the need; and never.

### Data Analysis

Quantitative study was conducted. The reliability of the questionnaire was checked with the coefficient of internal consistency Cronbach Alpha and the value was .896, indicating good reliability of the measurement for the variables in the scale. We checked the normality of the distribution of the variables with the coefficient of asymmetry (skewness) and flatness (kurtosis), which have the value in the interval between +1 and −1.^26^ In addition to the demographic data, we tried to assess how the teachers of the Faculty of Health Sciences follow the ergonomic principles of the environment. The data were analyzed using descriptive statistics, Pearson’s correlation coefficient, and factor analysis in SPSS Statistics 24. In the Options dialog box, we exclude cases list wise, that is, if a case has a missing value for a variable, it is excluded from the entire analysis.

## Results

### Sample Description

Only one-sixth (14%) of the participants were men, while 86% were women. The proportion of male teachers varied across the 8 departments of the faculty, depending on position and subject area. The age of faculty teachers ranged from 26 to 60 years. Most were between 36 and 45 years old (33%) and between 26 and 35 years old (31%). More than one-third (35%) of the participants had been in service for 11–20 years and 26% had been in service for less than 10 years.

### Physical Activities Break at Work

For teachers, several factors motivate them to take physical activities break at work. Surprisingly, 72% of teachers are familiar with the positive effects of taking an active break at work. When working with computers, 12% of teachers do not interrupt the work process until the process is complete. Only 7% of teachers knowingly and intentionally interrupt their work process every 2 hours while sitting to take an active break at work, which significantly affects productivity and health. 37% of teachers interrupt the work process due to physiological needs; 21% of teachers are forced to move due to pain or numbness.

The correlation analysis between the D: Back Pain and the other 2 variables was statistically significant at the .01 level. D: Back pain was statistically significantly associated with A: Time spent on the computer daily (*r* = .443, *ρ* < .01), with B: Physical activities break at work (*r* = .758, *ρ* < .01), and with C: Physical activity during leisure time (*r* = .574, *ρ* < .01). A positive and strong association exists between the variables B: Preventive rest at work and C: Physical activity in leisure time (*r* = −.800, *ρ* < .01). A medium statistical significant positive association exists between variables A: Time spent daily on the computer and B: Physical activities break at work (*r* = −.617, *ρ* < .01) and between variables A: Time spent daily on the computer and C: Physical activity in leisure time (*r* = −.560, *ρ* < .01). From the correlation analysis in Table 1, one can see the strength of the relationship between the variables.

**Table 1.** Results of Pearson Correlation Analysis for Back Pain.

Variables	*N*	Mean	SD	Pearson Correlation			
				A	B	C	D
A: Time spent daily on the computer	50	3.7	1.1	1	.617^a^	.560^a^	.443^a^
B: Physical activities break at work	50	3.5	1.1	.617^a^	1	.800^a^	.758^a^
C: Physical activity during leisure time	50	2.7	1.1	.560^a^	.800^a^	1	.574^a^
D: Back pain	50	3.6	1.3	.443^a^	.758^a^	.574^a^	1

Legend: Variables A, B, D evaluated frequency on scale from 1 to 5, variable C evaluated frequency on scale from 1 to 6.

^a^Correlation is significant at the level of .01.

### Professional Habits and Rules

The relationship between the dependent and independent variables was determined using regression analysis. The proportion of each variance of variables A: Time spent daily on the computer and B: Physical activities break at work in the total variance of the dependent variable D: Back pain. The results of the regression analysis are presented in Table 2.

**Table 2.** Results of the Linear Regression Analysis for Back Pain.

Variables	B*	Standard Error	*β* ^+^	*ρ*
A: Time spent daily on the computer	1.064	.068	.915	.000
B: Physical activities break at work	.756	.086	.786	.000
M: Age of employers	2.820	.525	.612	.000
N: Years of services	2.366	.419	.632	.000

Legend: B*= non-standardized coefficient, β^+^ = standardized multiple regression coefficient.

The results of the linear regression analysis showed that the dependent variable D: Back pain could be explained by 83.7% (*β*^+^ = .915; *ρ* = .000) fraction of total variability of the variable A: Time spent daily on the computer. The variable A: Time spent daily on the computer was defined as a variable of time, spent by teachers working with the computer for the purpose of work.

The results of the linear regression analysis explained 61.7% (*β*^+^ = .786; *ρ* = .000) of the total variability of variable D: Back pain with the variability of variable B: Physical activities break at work.

The results of the linear regression analysis showed that the dependent variable D: Back pain could be explained by 37.5% (*β*^+^ = .612; *ρ* = .000) fraction of total variability of the variable M: Age of employers where between the variables D: Back pain and M: Age of employers exists a medium statistical positive association (*r* = .612, *P* < .001).

The results of the linear regression analysis explained 39.9% (*β*^+^ = .632; *ρ* = .000) of the total variability of variables D: Back pain with the variability of variable N: Years of services, where between the variables also exists a medium statistical positive association.

### Influence of Habits

A reflection of the teacher’s personal status on the position of the various body parts that employees have when working with computers and D: Back pain is shown by the correlation analysis in Table 3.

**Table 3.** Results of Pearson Correlation Analysis for Back Pain and the Position of the Different Parts of the Body When Working With Computers.

Variables	*N*	Mean	SD	Pearson Correlation								
				D	E	F	G	H	I	J	K	L
D: Back pain	50	3.6	1.3	1	.411^b^	.315^a^	.446^b^	.483^b^	.669^b^	.766^b^	.577^b^	.608^b^
E: Sitting position	50	2.7	0.9	.411^b^	1	.405^b^	.725^b^	.457^b^	.538^b^	.584^b^	.543^b^	.692^b^
F: Body position	50	1.7	0.9	.315^a^	.405^b^	1	.381^b^	.435^b^	.419^b^	.531^b^	.499^b^	.595^b^
G: Head position	50	2.3	0.7	.669^b^	.538^b^	,419^b^	1	.561^b^	.451^b^	.852^b^	.778^b^	.577^b^
H: Shoulder position	50	2.3	0.7	.446^b^	.725^b^	.381^b^	.523^b^	1	.561^b^	.684^b^	.623^b^	.682^b^
I: Legs and feet position	50	1.6	0.9	.483^b^	.457^b^	.435^b^	.523^b^	.451^b^	1	.654^b^	.623^b^	.558^b^
J: Distance between eyes and screen	50	1.9	0.4	.766^b^	.584^b^	.531^b^	.654^b^	.654^b^	.852^b^	1	.839^b^	.662^b^
K: Screen inclination	50	2.6	0.5	.577^b^	.543^b^	.499^b^	.623^b^	.623^b^	.778^b^	.839^b^	1	.639^b^
L: Adjusting chair settings	50	1.8	0.4	.608^b^	.692^b^	.595^b^	.682^b^	.558^b^	.577^b^	.662^b^	.639^b^	1

Legend: Variables evaluated frequency on the scale from 1 to 5.

^a^Correlation is significant at the level of .05.

^b^Correlation is significant at the level of .01.

There is a statistically significant positive association between D: Back pain and the position of different parts of the body when using the computer.

With the help of factor analysis, we tried to find out the overall influence of position of different body parts while using computer on back pain among the teachers at Faculty of Health Sciences. Table 4 shows the factor loadings of the newly obtained factors. It can be seen that the eigenvalues of the selected factors and the variance rates of variable D: Back pain explained by individual factors have changed with rotation. The said variance rates are shown in Table 4.

**Table 4.** Rotated Factor Matrix for Two Factors of Back Pain.

Variables	Factor	
	The way of sitting	Characteristics of furniture
J: Distance between eyes and screen	.875	.372
K: Screen inclination	.856	.338
G: Head position	.837	.275
I: Legs and feet position	.640	.370
F: Body position	.536	.370
E: Sitting position	.269	.878
H: Shoulder position	.379	.804
L: Adjusting chair settings	.476	.743

Using the varimax method, we obtained the factor weights for 2 new factors, which are presented in Table 4 and account for 69.78% of the total variance of variable D: Back pain explained. A higher factor weight value means that the largest contribution to the variance belongs to the explanation of all changes in the disease. In the present case, the dimensions of the content of the first factor have high factor weights and explain the content of the first factor. The first factor, which after rotation accounted for 41.60% of the total variance of variable D: Back Pain explained, was “The way of sitting”. The second factor, which accounted for 28.18% of the total variance after rotation, was “Characteristics of furniture/workstation design".

## Discussion

With knowledge “For better public health” is the motto of the National Institute from Public Health,^27^ and there are many important reasons to highlight the workplace as an important arena for future health promotion.^13,24,28,29^ Musculoskeletal disorders are the most prevalent occupational diseases in Europe. About a quarter of European workers report that their work affects their health in the form of back pain. Similarly, muscle pain in the shoulders, neck, and/or upper/lower limbs is reported by about 22.8% of workers.^30^ Musculoskeletal disorders are a common occupational problem in the teaching profession.^4^ Our study confirms that back pain is one of the musculoskeletal disorders that occur in 6% of workers, while 13% of teachers report not having back pain. In addition to objective health status, workers' sick leave is influenced by many other factors from personal, professional, and wider environments,^4,28^ while performance patterns are already established as professional or personal habits and roles. In the Faculty of Health Sciences, 40% of faculty regularly exercise 3 to 5 times per week and lead healthy lifestyles. Working conditions have a major impact on absenteeism.^30^

We are not sufficiently aware that our bodies and our concentration benefit from regular breaks so that we can continue to work. From our research, 12% of employees never get up before completing their tasks, and 37% of employees get up due to physiological needs. The external causes of back pain occurrence pointed out by Zurc^20^ are usually due to workload, which she includes a mismatch between work furniture and anthropometric characteristics, as well as a lack of physical activity during leisure time. With the cited results, she points to the need for movement breaks (physical activity) at work before pain occurs. Using a meta-analysis, she showed a direct significant positive effect of physical activity in the workplace, which she included in the study. She highlighted 2 important forms of physical activity: leisure-time physical activity and workplace activities that have a synergistic effect. Leisure time physical activity^13,29,31^ and workplace physical activity^32^ have also been significantly associated with back pain in various studies of primary and secondary school teachers and health care workers.^33^

In our research, we found no statistically significant correlation between variables D: Back pain and C: Physical activity during leisure time, but a negative statistical correlation (*r* = −.460; *p* < .01) was found between variables C: Physical activity during leisure time and B: Physical activities break at work (Table 2). The factors that are negatively correlated with back pain are the protective factors mentioned by Erick and Smith.^4^

Physical activity is one of the healthy habits and an important part of leisure time. Only 5% of teachers in the Faculty of Health Sciences are not physically active in their free time, and 21% of teachers participate in sports activities only occasionally. In the study conducted by Biernat and Roguski^34^ in Poland, 43% of academic teachers chose physical activity as a way to spend their free time. Various authors present the importance of participation in recreational sports activities for academic teachers and confirm that a teacher’s lifestyle can influence the healthy lifestyle of students.^13,29,34–36^ This is one of the more important roles of the teaching profession. Technological advancement is most responsible for sedentary and passive lifestyle.^16,19^ To get information through our sedentary jobs, we often use information and communication technology. It is easier to receive information from a colleague via email or phone call than to interrupt sitting and intentionally, with the intention of taking a physical activity break at work, go to the office next door.

This behavior pattern does not prevent the occurrence of back pain and is not consistent with Zurc’s^19^ recommendations that physical activity supervised by an expert twice a week for 45 minutes during working hours is required to prevent back pain. Time spent daily on the computer work (*R*^2^ = .837; *P* < .01) and break for physical activity at work (*R*^2^ = .617; *P* < .01) represent an important part of the variance of the back pain variable, which is also confirmed by other authors.^11,32,35^

Age (*R*^2^ = .375; *P* < .01) and years of service (*R*^2^ = .399; *P* < .01) also account for an important part of the variance in back pain. In addition to the correlations between the variables, we also found the influence of these variables on back pain. In the study among public high school teachers in the city of Manila, no significant difference was found in age and gender between the group with back pain and the group without back pain (*P* < .05).^35^

Among the factors of back pain, several studies and research studies in the field of ergonomics^7,8,35,37-42^ cite the importance of ergonomic principles. Lunde^19^ highlights the importance of the workplace from the perspective of anatomy and physiology. Various authors^7,23,35,42-43^ showed a correlation between ergonomics and elements that also influence back pain.

Our study shows (Table 3) that the distance between the eyes and the monitor (*r* = .766; *P* < .05) is the element most strongly associated with back pain. The upper part of the monitor must be slightly farther from the eyes than the lower part. In our study, 57% of the teachers have their monitors in such a position, 4% of the teachers have the lower part of the monitor further away from the eyes, and this has a negative effect on neck posture. The importance of ergonomics and work design as a part of methodology is not only to increase productivity but also to improve the health and safety of employees, thus reducing the cost of teaching staff. In the 21st century, it is imperative that faculty consider both productivity issues and their efforts to improve the health and safety of their staff. Authors^44^ have found that it is worst when the monitor is below the eyes and facing downward.

The second element that has an important effect on back pain is the position of the head (*r* = .669; *P* < .05) with the eyes fixed on the monitor. According to the recommendations of Niebel and Freivalds,^44^ the monitor should be set 15° below eye level, which was confirmed by 39% of the teachers of the Faculty of Health Sciences.

The third element of variable position of different body parts during computer work that has a statistically significant association with back pain is the element of chair adjustment (*r* = .608; *P* < .05). In our study, 62% of teachers use chairs with hand rests. This is followed by the element of screen tilt (*r* = .577; *P* < .05). The fifth element is leg and foot position (*r* = .483; *P* < .05). In 57% of the teachers, the knees are at hip level and the feet are completely on the floor. Oyewole and his colleagues^45^ emphasized the importance of proportional height of the knees in relation to the height of the desk. Position of shoulders (*r* = .446; *P* < .05), which is the only one among the elements of position of different parts of the body whose correlation coefficient shows a statistically significant relationship. 12% of employees chose the sitting position with raised shoulders. The position of shoulders is most influenced by the position of the keyboard, which is placed in the middle of the desk for 76% of employees. Besides the position of the keyboard, the height of the chair also plays an important role,^25,37^ for 94% of the employees. According to the recommendation of Niebel and Freivalds,^44^ one should sit on a chair that is slightly bent backward, which supports the back better. In this position, the angle between the body and the legs is 90° or more. 34% of teachers use this position, which is more open and allows better blood circulation. The seventh element in our study that shows a statistically significant correlation with back pain is the sitting position (*r* = .411; *P* < .05) in relation to the seat. 71% of teachers reported sitting on two-thirds of the seat. The principles of ergonomic laws recommend^7,25,43^ that we should place most of our body weight on the seat when sitting. Some of the weight will also be on the floor, chair back, and forearms. Sitting all day can put pressure on the back and cause unwanted pain if the body parts are not adequately supported.^28,29^ Posture is the element that can be adjusted (*r* = .315; *P* < .01) with a suitable according to the recommendations of ergonomic principles.^7,25,43^ We believe that the Faculty of Health Sciences are not sufficiently aware of the importance of ergonomic recommendations. Only 57% of teachers have adjustable backs, and only 23% of teachers have the ability to adjust seat depth.

We wonder what influence the position of the different parts of the body during computer use has on back pain, so we performed a factor analysis. We obtained two new factors (Table 4) that explained 69.78% of the total variance of the variable back pain. According to the content of the items of the variable after rotation, we named the first factor “The way of sitting”. We called the second factor “Characteristics of furniture (workplace design)". With this result, we confirm that there is dependence between back pain and the position of different parts of the body during computer use in teachers. The goal of ergonomics science is to reduce the incidence of health problems among workers who use computers. Unfortunately, we have no information on whether employees who have an ergonomic chair take full advantage of these ergonomic opportunities. By supporting the body parts optimally, we reduce their static load. We reduce the negative effects of constant sitting by using ergonomic work chairs with appropriate lines that help the body to adopt a relaxed and correct posture. The additional possibility of adjusting the chair in height, depth, and rotation provides a better adaptation to each user and prevents discomfort, pain, or injury.

Working at a computer every day and ignoring ergonomic recommendations can lead to serious health problems. Any workplace design should follow the principles of modern ergonomics.^46^ We found that teachers are unaware of the consequences of sitting for long periods. In the Faculty of Health Sciences, 35% of the teaching staff have acquired knowledge about ergonomics during their training. 37% of the teaching staff have acquired knowledge in this field themselves.

For many years, many companies in Slovenia have been promoting the health of employees in various ways, namely, through seminars, workshops, and other forms of training. In practice, we can see that there are few companies that change their habits and introduce new roles when incorporating the field of ergonomics^18^ into their work environment and training. 36% of teachers in the Faculty of Health Sciences have acquired knowledge about ergonomics during their studies. 38% of them continue their education in this area on their own initiative. The results of the research conducted by Robertson and co-workers^40^ confirm that training employees in ergonomics and ergonomic adjustable chair helps to create an ergonomic workplace and increase employee productivity, which is a goal of every employer. Achievement of the objectives in the above studies has influenced the interest of employers in improving the ergonomic principles of the workplace. Increased productivity of individuals and positive impact on health status considering ergonomic adjustments of the workplace are confirmed by various authors^6,7,13,29,42^ in their studies. These findings support the initiative of teachers to change their work habits. The management of the Faculty of Health Sciences has offered a yoga retreat twice a week in the morning hours before work. Participants welcome the opportunity offered and notice positive effects of the yoga exercises. In addition to the above exercises, the Faculty of Health Sciences has fitness equipment available for use by teachers, but we did not find any particular enthusiasm among teachers for its use. Our findings contribute to the growing evidence for the long-term effectiveness of needs-based exercise counseling, as musculoskeletal disorders are still the most common cause of work disability among teachers worldwide^13^ and in Slovenia.^24^

## Limitations

Only one faculty member is participating in this study, but for health professionals like us, this study was very important.

We all sit too much during the day, even though we all know how important ergonomic principles are when working at a desk and during leisure time. As much as we need knowledge, we also need active breaks during our working hours and physical activities during our leisure time. Perhaps the method we used seems more for the convenience of the researchers than for the purpose of the study. We agree that measuring ergonomic measures and, for example, back pain cannot be accurately and effectively measured with a simple online questionnaire.

Limitatively, we find that the manuscript describes a single-domain study that does not present sufficiently original or unique results that are relevant to a regional or international audience. We got the answers we needed for our school leadership to take responsibility for teachers to change their work habits and support programs for staff such as physical activity in the workplace, morning yoga exercises before work, and some other sports such as cycling, badminton, skiing, or hiking that staff prefer and that are a good or possible way to prevent musculoskeletal disorders and absenteeism at work.

### Implications

The findings of this work may help raise awareness of health and safety in the workplace, in addition to clinical implications for ergonomists and therapists. The work may help therapists design a better workplace environment and/or recovery plan for workers with prolonged computer use.

The findings of this study present a challenge to managers and leaders in the Slovenian Education system to prepare intervention goals to improve teacher health, not only in institutions but also in crises, as was the case with COVID-19, when teachers stayed at home and worked online. The goals include redesigning the workplace to include ergonomic workplace design, changing work habits when physical activities interrupt the work process, and changing work roles so that teachers have fewer administrative tasks to complete in the future. The research was conducted prior to COVID-19. We now wonder if ergonomic principles were considered in the redesign of the workplace and how COVID-19 changed teachers' work habits and work roles.

## Conclusion

Sitting in front of the computer all day is a bad habit and a strain on the body. This fact can be confirmed by anyone who sits for hours in front of a screen. The human body has evolved into a shape that is best suited for slow movements. When using the computer, some parts of the body move little or are in an unsuitable position, especially when we use the mouse. Long-term computer use has negative effects on the health of individuals. Occasionally, pain occurs that we do not pay much attention to at first. Only when a chronic injury or even illness requires medical examination and long-term treatment do we become aware of the discomfort. With the above data, we would like to support the importance of pain research among teachers in education.

As health care professionals, we believe that it is possible to prevent many diseases related to back pain by developing good work habits and considering ergonomic principles when adapting the workplace, work processes, and work environment. Everyone involved in designing a workplace should be familiar with ergonomics, as only it can lead to improved well-being and increased productivity. We would like to emphasize that it is necessary to develop ergonomic promotion and intervention measures to maintain the health of workers in the work environment. These both preventive and curative projects have a good positive impact on students' health. Teachers and students need to be aware that there is a strong link between work roles, work habits, and training in the process of education.
